# Racial/Ethnic Disparities in Depressive Symptoms Among Pregnant Women Vary by Income and Neighborhood Poverty

**DOI:** 10.3934/publichealth.2015.3.411

**Published:** 2015-07-31

**Authors:** Catherine Cubbin, Katherine Heck, Tara Powell, Kristen Marchi, Paula Braveman

**Affiliations:** 1 School of Social Work, University of Texas at Austin, 1925 San Jacinto Blvd, D3500, Austin, TX 78712 USA; 2 Department of Family & Community Medicine, University of California, San Francisco, 3333 California Street, Suite 365, San Francisco, CA 94118 USA; 3 Current affiliation: School of Social Work, University of Illinois Champaign-Urbana, 1010 West Nevada Street, Urbana, IL 61801 USA

**Keywords:** race, ethnicity, income, neighborhood poverty, trajectory, depression

## Abstract

We examined racial/ethnic disparities in depressive symptoms during pregnancy among a population-based sample of childbearing women in California (N = 24,587). We hypothesized that these racial/ethnic disparities would be eliminated when comparing women with similar incomes and neighborhood poverty environments. Neighborhood poverty trajectory descriptions were linked with survey data measuring age, parity, race/ethnicity, marital status, education, income, and depressive symptoms. We constructed logistic regression models among the overall sample to examine both crude and adjusted racial/ethnic disparities in feeling depressed. Next, stratified adjusted logistic regression models were constructed to examine racial/ethnic disparities in feeling depressed among women of similar income levels living in similar neighborhood poverty environments. We found that racial/ethnic disparities in feeling depressed remained only among women who were not poor themselves and who lived in long-term moderate or low poverty neighborhoods.

## 1. Introduction

Approximately half of all women will experience a mental health problem at some point in their life time, with mood disorders such as depression among the leading mental health diagnoses [1]. Among pregnant women, recent estimates found that approximately 10–13% [2], [3] experience a major depressive episode or meet diagnostic criteria for depression, and up to 20% have reported depressive symptoms during pregnancy [4]. Consequences associated with prenatal depression include adverse birth outcomes, obstetric complications, fetal and neonatal well-being [5]–[8]. There are also known adverse health-related consequences and comorbidities associated with depression among women in general [9]–[13] and among pregnant women specifically, including lack of partner support, anxiety, stress, history of domestic violence, and alcohol consumption [14],[15].

Not all pregnant women have the same risk of experiencing prenatal depressive symptoms, however. Higher rates of depressive symptoms have been found among African American or Hispanic/Latina women compared with White women [16]–[18], and among women with low socioeconomic status (SES) in comparison to higher-SES women [19]–[23]. In addition, as the social ecological model [24] would predict, neighborhood-level factors, including both physical (e.g., decay/disorder, stable housing, traffic, noise, access to resources, transportation) and social characteristics (e.g., social cohesion and capital, violence, family structure, racial/ethnic concentration), can play a significant role in depression among diverse samples [25]–[28] and among pregnant women in particular [29],[30].

Given the large differences in individual-level SES and neighborhood environments according to race/ethnicity in the U.S., it is difficult to sort out their respective influences on depressive symptoms—as well as other health outcomes—via traditional regression approaches of controlling for them. Furthermore, increased attention on cross-level interactions, such as testing for differential effects of neighborhood factors according to individual-level SES factors and/or racial/ethnic group, could further understanding on which subgroups are more or less vulnerable to similar neighborhood environments. Methods to examine differential effects of neighborhood factors include statistical interaction and stratified analyses. While some existing neighborhood effects studies of depression have done this (for example, [31]–[33]), to our knowledge, no study has examined differential neighborhood effects on depressive symptoms among women during pregnancy, nor has any study stratified by individual-level SES, race/ethnicity, and neighborhood factors simultaneously.

In the current study, we examine racial/ethnic disparities in reported depressive symptoms during pregnancy among a population-based sample of childbearing women in California, first combined, and then simultaneously stratified by both an indicator of individual-level SES (family income) and neighborhood-level SES (long-term poverty history). Our hypothesis is that observed racial/ethnic disparities examined among women in combined, adjusted analyses will be non-existent or substantially eliminated in the stratified analyses. The rationale for our hypothesis is that structural inequalities are important upstream factors influencing depression, and pregnant women experiencing similar socioeconomic circumstances would be expected to be affected (as manifest by their depressive symptomatology) in a similar way, regardless of their race/ethnicity.

## 2. Materials and Methods

### 2.1. Data

For this study, we used data from the 2003-2010 California Maternal and Infant Health Assessment (MIHA), a collaborative project of the California Department of Public Health's Maternal, Child, and Adolescent Health Branch and researchers at the University of California, San Francisco. MIHA is an ongoing, statewide-representative survey of mothers delivering live infants in California during February through May annually, linked with birth certificate data and routinely geocoded based on mothers' residential address. Women are eligible to participate in MIHA if they are English- or Spanish-speaking California residents, aged 15 years or older, with singleton, twin, or triplet births, and have addresses recorded in birth certificates; the sample is selected according to region, education, and race/ethnicity, oversampling African Americans. Each February through May, during 2003–2009, MIHA interviewed 3,100 to 3,500 women representing over 150,000 births during those months. In 2010, the sample size increased to approximately 6,800. Self-administered surveys in English and Spanish were mailed to women 10–14 weeks after they have given birth, with reminder postcards and a second mailing to non-respondents. Telephone contact was attempted with women who had not responded and those whose surveys were returned because of incorrect addresses. During 2003–2010, among respondents, approximately 94% completed their surveys when the infant was between 2 and 7 months old (median 111 days). Questionnaires were completed by mail for 70% and by telephone for 30% of respondents; 72% of the surveys were completed in English and 28% in Spanish. Response rates exceeded 70% each year. Survey data were weighted to account for the sampling design and to reflect the population of births statewide for each year. The maternal characteristics of the MIHA sample were similar to those of all eligible births statewide (data not shown). The MIHA study was approved by the Institutional Review Boards at the University of California, San Francisco, and the California Department of Public Health; all participants gave informed consent.

The dependent variable was “feeling depressed during pregnancy,” defined as a “yes” response to the question: “During your pregnancy, did you ever have 2 weeks or longer when you felt sad, empty, or depressed for most of the day?” Other variables included age, parity, and race/ethnicity from the birth certificate, and marital status, education, and income from MIHA. Income was measured by self-report of previous calendar year's income from all sources (measured in 20 separate categories); it was then combined with family size and categorized into percent of the federal poverty level (< = 100%, 101–200%, 201–300%, 301–400%, or > 400%). Education was replaced with birth certificate reports where it was missing in MIHA (< 1%). Because women at the lowest ages would not have had the opportunity to complete their education, we restricted our analyses to those aged 22 years and over.

Based on our previous finding that neighborhood poverty histories are important to measure in relation to preterm birth, rather than only a cross-sectional poverty measure [34], we chose to use tract-level poverty rates from the Neighborhood Change Database [35] for 1970, 1980, 1990, and 2000, as well as from the American Community Survey for 2005–2009. We estimated poverty histories for every census tract in California using latent class growth modeling (LCGM). LCGM identifies distinct subgroups of the sample that follow a similar pattern of change over time on a given variable [36], [37], in this case, neighborhood poverty rates. The number of latent classes of neighborhood poverty was based on the Bayesian information criteria (BIC) value, the Lo, Mendell, and Rubin likelihood ratio test (LMR-LRT) statistic, and the entropy value. The LCGM method identified only three distinct latent classes of poverty histories, which we refer to as long-term low poverty, long-term moderate poverty, and long-term high poverty; it did not identify additional, more nuanced neighborhood classes, such as those with substantially increasing or decreasing poverty over time. All tracts were placed into the best-fitting of the three identified classes.

About 97% of birth certificate addresses geocoded accurately to a census tract. The final analytic dataset included 24,587 women with an accurate geocode and who identified as non-Hispanic African American, non-Hispanic Asian or Pacific Islander, Latina, or non-Hispanic White. These mothers lived in 6,608 census tracts in CA (out of 7,049 total), with an average of 3.7 mothers per tract (range 1–48). Approximately 81% of the census tracts had five or fewer sampled mothers, indicating little clustering by tract.

### 2.2. Analysis

We first examined frequency distributions of the variables stratified by race/ethnicity. We then examined the prevalence of feeling depressed, stratified by race/ethnicity, individual income (below vs. above poverty level), and neighborhood poverty history (three latent classes). Next, we constructed logistic regression models among the overall sample to examine both crude and adjusted racial/ethnic disparities in feeling depressed. Finally, adjusted logistic regression models were constructed, stratified by income and neighborhood poverty history, to examine racial/ethnic disparities in feeling depressed among women of similar income levels living in similar neighborhood poverty history areas. The LCGM was done using Mplus (version 6.12); all other analyses were done in SAS (version 9.3), taking into account the stratified sample design of MIHA and incorporating weights.

## 3. Results

Table 1 presents the individual- and neighborhood-level characteristics for the sample stratified by race/ethnicity (and nativity for Latina women). Asian/Pacific Islander and White women were older and gave birth to their first child in higher proportions than the other three groups; the proportion who were married or living with a partner was lowest among African American women. Socioeconomic differences among the racial/ethnic groups of women were apparent at the individual and neighborhood levels: Nearly 80% of immigrant Latinas had educational attainments that were at a high school graduate level or less, while over half of Asian/Pacific Islander and White women were college graduates; and while the majority of African American and Latina women had incomes below 200% of the poverty level, nearly half of Asian/Pacific Islander and White women had incomes that were over 400% of the poverty level. Relatively large proportions of African American and Latina women lived in neighborhoods characterized by long-term high poverty, while over 80% of Asian/Pacific Islander and White women were living in neighborhoods characterized by long-term low poverty. Finally, the “feeling depressed” prevalence rates varied from 17% for White women to double that for immigrant Latinas (34%).

**Table 1.** Sociodemographic characteristics, Maternal and Infant Health Assessment, 2003–2010, N = 24,587

	African American (n = 2981)		Asian/Pacific Islander (n = 2758)		Latina, immigrant (n = 7295)		Latina, US-born (n = 3827)		White (n = 7718)	
	n	%	n	%	n	%	n	%	n	%
*Maternal age*										
22–34 years	2413	(80.6)	2018	(72.7)	5905	(81.2)	3423	(88.3)	5809	(74.6)
35 years or older	569	(19.4)	740	(27.3)	1358	(18.8)	435	(11.7)	1917	(25.4)
*Parity*										
First birth	868	(29.2)	1278	(46.8)	1538	(21.8)	1114	(28.2)	3180	(40.9)
2–4 births	1834	(61.9)	1423	(51.4)	5181	(70.7)	2506	(66.0)	4315	(56.3)
5 or more births	278	(8.9)	57	(1.8)	539	(7.5)	237	(5.9)	227	(2.8)
*Marital status*										
Married/living together	1943	(64.5)	2597	(95.4)	6343	(88.1)	3161	(83.0)	7135	(93.7)
Single/Separated/Divorced	1015	(35.5)	151	(4.6)	853	(11.9)	681	(17.0)	555	(6.3)
*Maternal education*										
Less than high school	349	(9.8)	116	(3.3)	3062	(40.8)	545	(13.7)	326	(3.8)
High school graduate/GED	636	(21.7)	260	(8.6)	2645	(37.2)	963	(26.7)	856	(11.4)
Some college	1250	(43.1)	660	(22.6)	989	(14.6)	1589	(39.9)	2637	(33.2)
College graduate or more	730	(25.4)	1712	(65.5)	506	(7.3)	745	(19.8)	3884	(51.6)
*Family Income*										
Missing	199	(7.1)	201	(7.3)	919	(12.9)	183	(4.9)	370	(4.9)
< = 100% FPL	1168	(38.2)	398	(11.4)	4077	(55.1)	1203	(28.6)	1005	(11.2)
101–200% FPL	667	(22.7)	421	(14.4)	1681	(23.4)	1031	(27.5)	1185	(14.3)
201–300% FPL	302	(10.4)	275	(9.9)	285	(4.1)	491	(13.4)	951	(12.4)
301–400% FPL	190	(6.5)	281	(10.5)	115	(1.6)	322	(8.3)	874	(11.7)
> 400% FPL	456	(15.0)	1182	(46.4)	186	(2.8)	628	(17.2)	3341	(45.6)
*Neighborhood poverty trajectory*										
Long-term high poverty	454	(17.6)	108	(3.7)	1133	(17.2)	357	(9.6)	150	(2.0)
Long-term moderate poverty	966	(35.2)	459	(15.9)	3078	(43.6)	1299	(34.5)	1260	(16.5)
Long-term low poverty	1469	(47.2)	2130	(80.4)	2842	(39.2)	2062	(55.9)	6067	(81.5)
*Feeling depressed during pregnancy*										
% Yes	962	(31.4)	538	(19.0)	2403	(34.0)	1089	(29.5)	1290	(16.9)

FPL=federal poverty level

Table 2 presents the prevalence (or rates) of feeling depressed during pregnancy stratified by race/ethnicity, income (at or below vs. above 100% of the poverty level), and long-term neighborhood poverty history. Looking at the marginal (total) values, prevalence rates were higher for poor compared with non-poor women for each racial/ethnic group overall and for each neighborhood poverty history group overall. Patterns across neighborhood poverty histories were less clear, however, when examining prevalence rates within an income level for a particular racial/ethnic group. For example, among immigrant Latinas, the rates were stable across neighborhood poverty histories for poor women; for non-poor immigrant Latinas, those living in low poverty neighborhoods had lower rates of depressive symptoms than women in poorer neighborhoods. Only among White women and non-poor Asian/Pacific Islanders were the patterns consistent. The lowest prevalence rate overall was among non-poor White women living in long-term low poverty neighborhoods (14%) and the highest rate was among poor White women living in long-term high poverty neighborhoods (52%).

Table 3 presents the unadjusted model and fully adjusted logistic regression model for feeling depressed among the combined sample. In the unadjusted model, greater odds were observed for each of the other racial/ethnic groups compared with White women, which were attenuated in the adjusted model (except for Asian/Pacific Islanders). Parity, marital status, education and income remained statistically significant but attenuated in the adjusted model as well. Greater odds were observed for long-term high and moderate poverty histories compared with long-term low poverty in the unadjusted model, and those odds were reduced to non-significance in the adjusted model, as is often observed in neighborhood effects analyses.

Table 4 presents adjusted logistic regression models, similar to Table 3, but now stratified by income and neighborhood poverty history. The main finding is that significant racial/ethnic disparities were, for the most part, restricted to women who were more socioeconomically advantaged in terms of their own incomes and the poverty histories of their neighborhoods of residence. Poor women of color, and non-poor women of color in long-term high poverty neighborhoods, experienced similar odds of feeling depressed during pregnancy as White women. In contrast, non-poor women of color in long-term moderate or low poverty neighborhoods experienced odds that were 40-80% higher than White women.

**Table 2.** Feeling depressed during pregnancy by individual- and neighborhood-level poverty trajectory, Maternal and Infant Health Assessment, 2003–2010, N = 21,914*

	Long-term high poverty			Long-term moderate poverty			Long-term low poverty			Total	
	%			%			%			%	
**African American**											
< = 100% FPL	45.8			36.5			38.7			39.7	
> 100% FPL	29.1			31.3			23.0			26.3	
**Asian/Pacific Islander**											
< = 100% FPL	16.0			36.7			36.2			33.9	
> 100% FPL	29.0			19.3			16.5			17.2	
**Latina, immigrant**											
< = 100% FPL	37.0			38.4			38.2			38.1	
> 100% FPL	30.9			32.0			26.4			29.1	
**Latina, US-born**											
< = 100% FPL	42.8			46.3			41.0			43.6	
> 100% FPL	24.6			29.2			21.2			23.8	
**White**											
< = 100% FPL	51.7			37.0			35.3			36.7	
> 100% FPL	20.8			16.6			13.8			14.3	
**Total**											
< = 100% FPL	38.2			39.4			38.0			38.7	
> 100% FPL	27.4			25.3			17.1			19.3	

*Excludes women with missing income

FPL = federal poverty level

**Table 3.** Logistic regression models for feeling depressed during pregnancy, Maternal and Infant Health Assessment, 2003–2010, N = 24,587

	Unadjusted		Adjusted	
	OR	(95% CI)	OR	(95% CI)
*Race/ethnicity*				
African American	2.25***	(2.01–2.52)	1.30***	(1.14–1.47)
Asian/Pacific Islander	1.16*	(1.02–1.31)	1.28***	(1.13–1.46)
Latina, immigrant	2.53***	(2.33–2.75)	1.36***	(1.22–1.52)
Latina, US-born	2.06***	(1.86–2.28)	1.36***	(1.22–1.53)
White	1.00		1.00	
*Maternal age*				
22–34 years	1.00		1.00	
35 years or older	0.79***	(0.72–0.86)	0.94	(0.86–1.04)
*Parity*				
First birth	0.68***	(0.63–0.73)	0.86***	(0.79–0.93)
2-4 births	1.00		1.00	
5 or more births	1.91***	(1.66–2.19)	1.39***	(1.20–1.61)
*Marital status*				
Married/living as married	1.00		1.00	
Single/Separated/Divorced	2.41***	(2.20–2.64)	1.66***	(1.49–1.84)
*Maternal education*				
Less than high school	3.49***	(3.16–3.86)	1.38***	(1.20–1.59)
High school graduate/GED	2.71***	(2.46–2.98)	1.25***	(1.10–1.41)
Some college	2.10***	(1.92–2.31)	1.33***	(1.19–1.48)
College graduate or more	1.00		1.00	
*Income*				
Missing	2.44***	(2.11–2.83)	1.67***	(1.42–1.97)
< = 100% FPL	4.84***	(4.37–5.37)	2.84***	(2.47–3.26)
101–200% FPL	3.24***	(2.90–3.62)	2.20***	(1.92–2.52)
201–300% FPL	2.06***	(1.79–2.36)	1.66***	(1.43–1.93)
301–400% FPL	1.62***	(1.38–1.90)	1.43***	(1.21–1.68)
> 400% FPL	1.00		1.00	
Neighborhood poverty trajectory				
Long-term high poverty	1.89***	(1.68–2.11)	0.98	(0.87–1.12)
Long-term moderate poverty	1.71***	(1.59–1.84)	1.07	(0.99–1.16)
Long-term low poverty	1.00		1.00	

*p < 0.05; **p < 0.01; ***p < 0.001

**Table 4.** Racial/ethnic disparities in feeling depressed during pregnancy, Maternal and Infant Health Assessment, 2003–2010, N = 21,914

Neighborhood Poverty Trajectory	Race/Ethnicity	< = 100% FPL		> 100% FPL	
		OR	(95% CI)	OR	(95% CI)
Long-term high poverty	African American	0.74	(0.34–1.58)	1.42	(0.65–3.09)
	Asian/Pacific Islander	0.16**	(0.05–0.50)	1.52	(0.53–4.39)
	Latina immigrant	0.54	(0.27–1.09)	1.77	(0.82–3.81)
	Latina US born	0.67	(0.32–1.43)	1.13	(0.53–2.44)
White	1.00		1.00	
Long-term moderate poverty	African American	0.93	(0.64–1.34)	1.78***	(1.29–2.44)
	Asian/Pacific Islander	1.14	(0.68–1.90)	1.34	(0.92–1.95)
	Latina immigrant	1.13	(0.83–1.55)	1.77***	(1.33–2.35)
	Latina US born	1.46*	(1.02–2.08)	1.60***	(1.21–2.11)
White	1.00		1.00	
Long-term lowpoverty	African American	0.96	(0.69–1.32)	1.52***	(1.25–1.86)
	Asian/Pacific Islander	1.19	(0.80–1.78)	1.39***	(1.19–1.64)
	Latina immigrant	1.28	(0.99–1.66)	1.61***	(1.33–1.96)
	Latina US born	1.21	(0.89–1.64)	1.39***	(1.18–1.64)
	White	1.00		1.00

Models adjusted for age, parity, marital status, and maternal education; excludes women with missing income. *p < 0.05; **p < 0.01; ***p < 0.001

## 4. Discussion

Our hypothesis was partially supported. On the one hand, we did find in the combined sample that racial/ethnic disparities in feeling depressed during pregnancy were essentially eliminated for women who were poor (regardless of their neighborhood poverty history) or women who were not poor but living in the most socioeconomically disadvantaged neighborhoods (i.e., those experiencing long-term high poverty concentration). On the other hand, the substantial and highly significant racial/ethnic disparities in feeling depressed in the adjusted analysis for the sample combined were not eliminated in the stratified analyses. Instead, in the stratified analyses, racial/ethnic disparities in feeling depressed during pregnancy remained among women who were not poor themselves and who were living in long-term moderate or low poverty neighborhoods (i.e., women who were more socioeconomically advantaged). To our knowledge, this is the first study to examine racial/ethnic disparities in a health-related outcome stratifying simultaneously by individual- and neighborhood-level poverty status while adjusting for potential confounders.

In contrast to some other research [25]–[30], we did not find that an indicator of neighborhood socioeconomic status was significantly associated with depressive symptoms after accounting for demographic and socioeconomic differences in the characteristics of the women residing within them. This may be due to individual-level income and educational attainment being a mediating factor on the pathway between neighborhood poverty history and depressive symptoms and thus our adjusted model may represent an over-adjustment. Also, Mair et al. [27] found that more specific features of neighborhoods, such as the built environment and social processes, were more consistently associated with depression-related outcomes compared with socioeconomic measures of neighborhoods, such as we used here.

The results of the stratified analyses suggest that non-poor women of color living in more advantaged neighborhoods are not enjoying the same mental health advantages compared with their White counterparts (40–80% higher odds of feeling depressed). This suggests several possible mechanisms. Women of color may feel they have a lower relative social standing or feel relatively deprived compared with their White counterparts, and/or may be more isolated in their communities. As well, women of color in more socioeconomically advantaged circumstances may paradoxically experience more discrimination, chronic vigilance in anticipation of discrimination, and/or feel a greater need to prove themselves, because they may be moving in a predominantly White sphere [38]–[43]; this could contribute to depressive feelings. Research on other health outcomes, such as preterm birth and infant mortality, suggests that the Black/White disparity *increases* with increasing socioeconomic attainment [43]–[48].

In addition, although the findings were not statistically significant, women of color in the most socioeconomically disadvantaged circumstances (poor themselves and living in long-term high poverty neighborhoods) appeared to have a pattern of lower odds of depressive symptoms compared with their White counterparts, suggesting that they may be more resilient—at least with respect to affective mental health—to challenging economic circumstances compared with their White counterparts. The highest prevalence of depressive symptoms among any group in the study was among poor White women living in long-term high poverty neighborhoods (52%), although the prevalence for African American women in those neighborhoods was also quite high (46%). Moreover, poor women overall had a twofold higher prevalence of depressive symptoms compared with non-poor women (39 vs. 19%), and women in neighborhoods that have experienced long-term high poverty also had high prevalences (38% among poor women and 27% among non-poor women). These socioeconomic inequalities in mental health should not be overlooked, especially given the population sizes that are affected (nearly two-fifths of women were poor and nearly one-fifth lived in neighborhoods with long-term high poverty) as well as the myriad harmful effects of poverty.

Several limitations of this study deserve mention. Neighborhoods are based on census boundaries, which is the only feasible option using a large, population based data set such as MIHA. However, census tract boundaries, although initially designed to contain homogenous places with logical boundaries (e.g., a major street or stream on one side of a boundary), may not conform precisely to the geography of a neighborhood nor women's perceptions of their neighborhoods. As well, mobility is high during pregnancy; therefore, the census tract at the time of birth may not represent the same type of neighborhood throughout her pregnancy. However, our unpublished data have revealed that women who move soon after birth (2–7 months later when the MIHA survey is completed) are moving, by and large, to very similar types of neighborhoods [49]. Given that we were stratifying by both individual- and neighborhood-level poverty, a cutoff of below vs. above poverty-level for individual-level income was necessary to maintain adequate cell sizes. Women with incomes above the poverty level could have relatively low or relatively high incomes, and women of color (aside from Asian/Pacific Islanders) were likely to have been concentrated at the lower end of the income range, thus perhaps accounting for their higher odds compared with White women in the stratified models. Cross-sectional data precludes making causal inferences, and selection into neighborhoods cannot be ruled out as an alternative explanation.

In terms of measurement, the single, self-reported item on depressive symptoms during pregnancy is not equivalent to a clinical diagnosis of depression, and reporting these symptoms may represent a relatively minor episode in a pregnant woman's life. However, the Behavioral Risk Factor Surveillance System (BRFSS) from 2008 data reported that 21% of women overall had a diagnosis of depression from a health care professional in her lifetime [50], which is in line with our findings of 17–34% of pregnant women reporting depressive symptoms (depending on race/ethnicity). These values are higher than what other surveys have found with different populations, measurement instruments, and/or survey administration. For example, the state-based Pregnancy Risk Assessment Monitoring System from found that 11% of women of reproductive age reported pre-pregnancy depression (2009) and 9.5% reported post-partum depression (2011) based on single items [50]–[51]. Differences with MIHA estimates of depressive symptoms during pregnancy may be due to differences in the populations (e.g., PRAMS states are likely to be comprised of higher SES women on average compared with MIHA). Nationally-representative surveys (Behavioral Risk Factor Surveillance System [2008], National Health and Nutrition Examination Survey [2005–2008]) using the Patient Health Questionnaire reported between 8–12% of women with moderate or severe depressive symptoms [50]. The Kessler-6 and Kessler-10 are also used in population-based surveys, including the California Health Interview Survey and the National Health Interview Survey (in which 4–8% of women reported serious psychological distress) [50], [52]. Similarly, the National Comorbidity Survey found that 7% of adults had a major depressive disorder in the past year based on the Composite International Diagnostic Interview [53]. There may also have been racial/ethnic or cultural differences in reporting depressive symptoms in the MIHA survey (i.e., lack of measurement equivalence) and we did not have information on depression treatment; access to and cultural acceptance of formal mental health care services—and thus self-reported depressive symptoms—are likely to vary among pregnant women according to race/ethnicity and/or socioeconomic status.

Despite these limitations—many of which exist in most neighborhood effects research—our findings are strengthened by using a large, ethnically diverse, statewide-representative sample of women in California, where one in 8 births in the U.S. occurs. In addition, rather than relying upon a static measure of neighborhood poverty, we calculated longitudinal poverty histories, based on knowledge suggesting that historical experiences of neighborhoods should be taken into account. Finally, the main strength of our study is the examination of racial/ethnic disparities for women living in similar economic circumstances in terms of both their own income and the poverty trajectory of their neighborhoods.

In conclusion, we found racial/ethnic disparities in depressive symptoms during pregnancy only among relatively more socioeconomically advantaged women, taking into account both their own income levels and the poverty experiences of their neighborhoods, both factors known to be associated with depression. Disparities between women of color and White women were essentially eliminated among poor women, after adjustment for age, parity, marital status, and maternal education, regardless of the poverty experience of their neighborhoods. Although poor women continued to suffer higher levels of mental health problems compared to their non-poor counterparts, our findings suggest that non-poor women of color living in neighborhoods without the highest poverty levels may be a vulnerable population in need of increased attention and intervention. Further research, perhaps best conducted through qualitative approaches, is needed to identify potential mechanisms leading to higher odds of depressive symptoms for these pregnant women, such as relative deprivation, perceived low social standing, social isolation, and/or discrimination.

## References

[1] Kessler RC, Berglund P, Demler O (2005). Lifetime prevalence and age-of-onset distributions of DSM-IV disorders in the National Comorbidity Survey Replication. Arch Gen Psychiatry.

[2] Gaynes BN, Gavin N, Meltzer-Brody S (2005). Perinatal depression: prevalence, screening accuracy, and screening outcomes. Evid Rep Technol Assess (summary).

[3] Melville JL, Gavin A, Guo Y (2010). Depressive disorders during pregnancy: prevalence and risk factors in a large urban sample. Obstet Gynecol.

[4] Bowen A, Muhajarine N (2006). Antenatal depression. Can Nurs.

[5] Alder J, Fink N, Bitzer J (2007). Depression and anxiety during pregnancy: a risk factor for obstetric, fetal and neonatal outcome? A critical review of the literature. J Matern Fetal Neonatal Med.

[6] Grote NK, Bridge JA, Gavin AR (2010). A meta-analysis of depression during pregnancy and the risk of preterm birth, low birth weight, and intrauterine growth restriction. Arch Gen Psychiatry.

[7] Dayan J, Creveuil C, Marks MN (2006). Prenatal depression, prenatal anxiety, and spontaneous preterm birth: a prospective cohort study among women with early and regular care. Psychosom Med.

[8] Diego MA, Field T, Hernandez-Reif M (2009). Prenatal depression restricts fetal growth. Early Hum Dev.

[9] Murray CJ, Lopez AD, Murray CJ, Lopez AD (1994). Alternative visions of the future: projecting mortality and disability, 1990-2020. The Global Burden of Disease: A Comprehensive Assessment of Mortality and Disability from Disease, Injuries, and Risk Factors in 1990 and Projected to 2020.

[10] Salomon JA, Vas T, Hogan DR (2012). Common values in assessing health outcomes from disease and injury: disability weights measurement study for the Global Burden of Disease Study 2010. Lancet.

[11] Korstein SG, Schatzberg AF, Thase ME (2000). Gender differences in chronic major and double depression. J Affect Disord.

[12] Pajer K (1995). New Strategies in the Treatment of Depression in Women. J Clin Psychiatry.

[13] Lindgren K (2001). Relationships among maternal–fetal attachment, prenatal depression, and health practices in pregnancy. Res Nurs Health.

[14] Marcus SM, Flynn HA, Blow FC (2003). Depressive symptoms among pregnant women screened in obstetrics settings. J Womens Health.

[15] Lancaster C, Gold KJ, Flynn HA (2010). Risk factors for depressive symptoms during pregnancy: a systematic review. Am J Obstet Gynecol.

[16] Orr ST, Blazer DG, James SA (2006). Racial disparities in elevated prenatal depressive symptoms among black and white women in eastern North Carolina. Ann Epidemiol.

[17] Canady RB, Bullen BL, Holzman C (2008). Discrimination and symptoms of depression in pregnancy among African American and White women. Womens Health Issues.

[18] Rich-Edwards JW, Kleinman K, Abrams A (2006). Sociodemographic predictors of antenatal and postpartum depressive symptoms among women in a medical group practice. J Epidemiol Community Health.

[19] Leigh B, Milgrom J (2008). Risk factors for antenatal depression, postnatal depression and parenting stress. BMC Psychiatry.

[20] Westdahl C, Milan S, Magriples U (2007). Social support and social conflict as predictors of prenatal depression. Obstet Gynecol.

[21] Groffen DA, Koster A, Bosma H (2013). Unhealthy lifestyles do not mediate the relationship between socioeconomic status and incident depressive symptoms: The Health ABC study. Am J Geriatr Psychiatry.

[22] Lorant V, Deliege D, Eaton W (2003). Socioeconomic inequalities in depression: a meta-analysis. Am J Epidemiol.

[23] Huurre T, Eerola M, Rahkonen O (2007). Does social support affect the relationship between socioeconomic status and depression? A longitudinal study from adolescence to adulthood. J Affect Disord.

[24] McLeroy KR, Bibeau D, Steckler A (1998). An ecological perspective on health promotion programs. Health Educ Q, Winter.

[25] Diez-Roux AV, Mair C (2010). Neighborhoods and health. Ann N Y Acad Sci.

[26] Truong KD, Ma S (2006). A systemic review of relations between neighborhoods and mental health. J Ment Health Policy Econ.

[27] Mair C, Diez-Roux AV, Galea S (2008). Are neighbourhood characteristics associated with depressive symptons? A review of evidence. J Epidemiol Community Health.

[28] Kim D (2008). Blues from the neighborhood? Neighborhood characteristics and depression. Epidemiol Rev.

[29] Messer LC, Maxson P, Miranda ML (2012). The urban built environment and associations with women's psychosocial health. J Urban Health.

[30] Giurgescu C, Misra DP, Sealy-Jefferson S (2015). The impact of neighborhood quality, perceived stress, and social support on depressive symptoms during pregnancy in African American women. Soc Sci Med.

[31] Gutman LM, Sameroff AJ (2004). Continuities in depression from adolescence to young adulthood: contrasting ecological influences. Dev Psychopathol.

[32] Gary TL, Stark SA, LaVeist TA (2007). Neighborhood characteristics and mental health among African American and Whites living in a racially integrated urban community. Health Place.

[33] Weich S, Twigg L, Holt G (2003). Contextual risk factors for the common mental disorders in Britain: a multilevel investigation of the effects of place. J Epidemiol Community Health.

[34] Margerison-Zilko C, Cubbin C, Jun J (2015). Beyond the cross-sectional: neighborhood poverty histories and preterm birth. Am J Public Health.

[35] Tatian PA Neighborhood Change Database (NCDB) 1970-2000 Tract data: data user's guide long form release.

[36] Andruff H, Carraro N, Thompson A (2009). Latent class growth modelling: a tutorial. Tutor Quant Methods Psychol.

[37] Nagin DS, Tremblay RE (2001). Analyzing developmental trajectories of distinct but related behaviors: A group-based method. Psychol Methods.

[38] Hudson DL, Neighbors HW, Geronimus AT (2012). The relationship between socioeconomic position and depression among a US nationally representative sample of African Americans. Soc Psychiatry Psychiatr Epidemiol.

[39] Nuru-Jeter A, Dominguez TP, Hammond WP (2009). “It's the skin you're in”: African-American women talk about their experiences of racism. An exploratory study to develop measures of racism for birth outcome studies. Matern Child Health J.

[40] Cole ER, Omari SR (2003). Race, class and the dilemmas of upward mobility for African Americans. J Soc Issues.

[41] Smedley BD (2012). The lived experience of race and its health consequences. Am J Public Health.

[42] Dailey AB, Kasl SV, Holford TR (2010). Neighborhood- and individual-level socioeconomic variation in perceptions of racial discrimination. Ethn Health.

[43] Braveman PA, Heck K, Egerter S (2015). The role of socioeconomic factors in black-white disparities in preterm birth. Am J Public Health.

[44] Jackson FM, Phillips MT, Hogue CJ (2001). Examining the burdens of gendered racism: implications for pregnancy outcomes among college-educated African American women. Matern Child Health J.

[45] Collins JW, Rankin KM, David RJ (2011). African American women's lifetime upward economic mobility and preterm birth: the effect of fetal programming. Am J Public Health.

[46] Mustillo S, Krieger N, Gunderson EP (2004). Self-reported experiences of racial discrimination and black-white differences in preterm and low-birthweight deliveries: The CARDIA Study. Am J Public Health.

[47] Kramer MR, Cooper HL, Drews-Botsch CD (2010). Metropolitan isolation segregation and black-white disparities in very preterm birth: a test of mediating pathways and variance explained. Soc Sci Med.

[48] Kramer MR, Hogue CR (2008). Place matters: variation in the black/white very preterm birth rate across U.S. metropolitan areas, 2002–2004. Public Health Rep.

[49] Margerison-Zilko C, Cubbin C, Jun J (in press). Post-partum residential mobility among a statewide representative sample of California women, 2003-2007. Matern Child Health J.

[50] Reeves WC, Strine TW, Pratt LA (2011). Mental illness surveillance among adults in the United States. MMWR.

[51] Robbins CL, Zapata LB, Farr SL (2014). Core state preconception health indicators – Pregnancy Risk Assessment Monitoring System and Behavioral Risk Factor Surveillance System, 2009. MMWR.

[52] Zahnd E, Wyn R (2012). Over one million adult women in california report serious psychological distress during the past year.

[53] Kessler RC, Berglund P, Demler O (2003). The epidemiology of major depressive disorder: results from the National Comorbidity Survey Replication (NCS-R). JAMA.

